# From art to health action: lessons from a community-based, culturally tailored arts-meets-health educational campaign in Hispanic communities

**DOI:** 10.3389/fpubh.2024.1385916

**Published:** 2024-04-12

**Authors:** Thomas J. Urich, Woori Lee, Justine Po, Arthur Bookstein, Rosa Barahona, Lourdes Baezconde-Garbanati

**Affiliations:** ^1^Dana and David Dornsife College of Letters, Arts and Sciences, University of Southern California, Los Angeles, CA, United States; ^2^Georgetown University School of Medicine, Washington, DC, United States; ^3^Department of Population and Public Health Sciences, Keck School of Medicine of University of Southern California, Los Angeles, CA, United States

**Keywords:** COVID-19, Hispanic, community, Los Angeles, art, outreach, mitigation behaviors, art meets public health

## Abstract

**Introduction:**

Robust digital and community-led approaches are needed to combat health misinformation, as highlighted by the COVID-19 pandemic. Such gaps in public health outreach, compounded by systemic health barriers, contributed to higher rates of COVID-19 infection, mortality, and mental health effects among Hispanics during the peak of the pandemic. Thus, we conducted a community-based art-meets-health intervention [Stay Connected Los Angeles (SCLA)] to address the impacts of the COVID-19 pandemic in Hispanic communities.

**Methods:**

Led by local artists in collaboration with public health specialists and community members, SCLA used multimedia to promote infection mitigation behaviors and psychological well-being among the 120,000 residents of Eastern Los Angeles. Campaign materials were designed with input from community representatives and included digital media, large-scale murals, and comic-book style pieces. Two semi-structured focus groups (one in English and another in Spanish) were conducted to solicit participants’ views on attributes of the campaign. Independent coders analyzed transcripts and applied thematic analysis to summarize key learnings regarding central health and mitigation messages, media modalities, how health information would be communicated, and the ideal spokespersons for delivering health-related messages.

**Results:**

Focus group participants emphasized the effectiveness of social media, GIFs, and references to popular media. Further, youth involvement in the creative process was deemed to be important. Participants highlighted the need for clarity in public health messaging and adaptation of visual campaigns to the preferences of diverse age groups through different art styles. Finally, community leaders were found to be critical health information sources.

**Discussion:**

As a model of a culturally tailored arts-meets health public education campaign, SCLA yielded valuable information on how to structure future public health messaging and media to create a meaningful improvement in health knowledge, mental well-being, and compliance with mitigation behaviors in communities that are often overlooked. Contributions from local artists can heighten appeal and acceptability of messages.

## 1 Introduction

Times of crisis often bring inequity into the spotlight, highlighting the need for multidisciplinary solutions. The coronavirus disease of 2019 (COVID-19) pandemic was one such crisis that made health inequities markedly apparent. During the height of the pandemic, a study in Los Angeles of over 500,000 COVID-19 test results determined that people identifying as Hispanic tested positive for COVID-19 at rates over three times higher than people who did not identify as Hispanic (1). Further, Hispanics in Los Angeles had a COVID-19 mortality rate of 192 per 100,000 persons, compared to 119 for non-Hispanic Black individuals and 69 for non-Hispanic White individuals, respectively (2). A study on the mental health impacts of the pandemic also found that individuals identifying as Hispanic were 10 times more likely to meet diagnostic criteria for depression due to noted exposure, isolation, and cumulative burden (3). In light of disparities in these physical and mental health domains, the Stay Connected Los Angeles (SCLA) campaign was developed as a public health campaign combining community-academic partnerships, as well as digital and traditional arts and design.

From a systemic lens, many structural and social determinants of health predispose Hispanics to increased risk of contracting COVID-19. Hispanics are more likely to live in crowded households, use public transportation, and work in professions that require in-person attendance, such as custodial work or retail professions (4). Furthermore, historical medical racism and exploitation, language barriers, and lack of representation in COVID-19 outreach and clinical trials have contributed to vaccine hesitancy and misinformation within Hispanic communities (4).

Owing to the social and historical factors above, there are several complexities in improving preventive health behaviors in Hispanic communities. Previous research by our group and others has identified that addressing systemic healthcare access issues, while critical, is not sufficient to overcome health barriers in this population that are also rooted in cultural beliefs, knowledge gaps, and distrust in the healthcare system (5, 6). These barriers have implications for addressing future pandemics, emergent or recurrent diseases, and complex seasonal respiratory infections (Influenza, RSV, COVID-19), as vaccination and mitigation behaviors directly impact disease burden. In contrast to the need for health education approaches that promote communal trust, cultural awareness, and meet individual literacy needs, public health campaigns often lack Spanish language or low literacy components, and do not involve community members in their development (7). Further, digital and social media approaches to health education represent a critical yet underutilized strategy, as nearly 75% of internet users search for health-related information online (8). Implementing social media as one of multiple approaches to health education removes physical barriers to health resources, while meeting community members in the digital spaces they are already using (9). These approaches have demonstrated efficacy in reaching traditionally underserved groups, including youth, older individuals, and those with lower socioeconomic status (10). Artistic expression has also been found to improve mental and physical well-being (11). A previous intervention in Australia focused on the use of a traveling art exhibit for mental health promotion, demonstrating reported reduced feelings of mental health stigma, increased social connection, and increased subjective well-being among both artists and viewers (12).

To address these gaps in health education, Stay Connected Los Angeles (SCLA) was designed as a community-based, culturally tailored arts-meets-health educational campaign for the Eastern part of Los Angeles. Eastern Los Angeles (Boyle Heights, Lincoln Heights, El Sereno) is home to a vibrant Hispanic community, with individuals of Hispanic descent making up 95.5% of the total population (13). The goal of this project was to determine the utility of a community-based campaign that included local artists and centered upon health promotion and disease prevention strategies, utilizing community based participatory principles. The goal was to increase health knowledge, encourage mitigation behaviors, and address mental well-being during the COVID-19 pandemic.

## 2 Materials and methods

A total of 55 bi-weekly project meetings, 14 Community Advisory Board meetings, two focus groups with Spanish-speaking and English-speaking participants (*n* = 24), and a townhall meeting with 46 community residents provided data for this campaign. All participants were local residents of the Eastern Los Angeles area, specifically in the neighborhoods of Boyle Heights, El Sereno, and Lincoln Heights. Materials were reviewed during Community Advisory Board meetings on a monthly basis as progress was made on various art pieces. Details on the campaign design, community-based participatory approach, and thematic analysis are presented below. Full IRB approval from the University of Southern California was obtained for this study.

### 2.1 Genesis of the campaign

The SCLA focused on Hispanic sub-populations particularly vulnerable to contracting COVID-19, such as workers who were unable to work from home, multigenerational households, people using public transportation, immigrants, and the older adult. This artistic-driven health education campaign created culturally tailored artwork to provide important information regarding health safety behaviors such as handwashing, masking, and physical distancing. Another goal was to increase social connection to support and mental well-being for participants during a health crisis of the magnitude of the COVID-19 pandemic. Detailed information on SCLA campaign elements can be found in the Journal of Arts and Science Research (14).

### 2.2 Community based participatory research

From the start, this project emphasized the importance of the active involvement and leadership of community members. Utilizing community based participatory strategies and principles (15), the community advisory committee established by SCLA allowed for regular joint decision making among project leaders (in art and health) and community members to be made throughout the entire campaign. It provided opportunities for mutual learning to occur from both project leaders and community members. The artists who created works for this campaign also had a direct connection to a team composed of public health experts and campaign coordinators, further enabling a strong connection to be made with the community. Our usage of community based participatory principles allowed us to better target our communities of interest and create health promoting art pieces with imaging and messaging that were both culturally relevant and meaningful.

### 2.3 Campaign content

Eleven artists employed a wide variety of media platforms in the pieces that they created, such as murals, billboards, comic strips, spoken word, theater performances, and youth paintings. The artists also created multiple styles and forms of artwork ranging from GIFs to comic book style pieces. Each art piece that was put out for participants to view all centered upon common health promotion and disease preventing practices such as masking and distancing and are available through the Stay Connected L.A. website.^1^

### 2.4 Evaluation and analysis

Two focus groups were used to elicit participant feedback (*n* = 24) regarding initial perceptions and feelings toward the art pieces. Participants were asked to share their thoughts and impressions for each of the art pieces, and also voted for which art piece they wanted to be the primary work displayed in the campaign. Participants also suggested ways to improve the pieces and their ability to convey messaging surrounding health-promoting behaviors. Upon completion of these focus groups, two independent coders used ATLAS TI to pull important themes and quotes from transcripts. All information was available in Spanish and English. Below we present findings from these focus groups, sharing descriptive and thematic analyses of four major art pieces utilized for determining final campaign elements.

## 3 Results

### 3.1 Figures—photos of the campaign artwork

**Figure 1:**
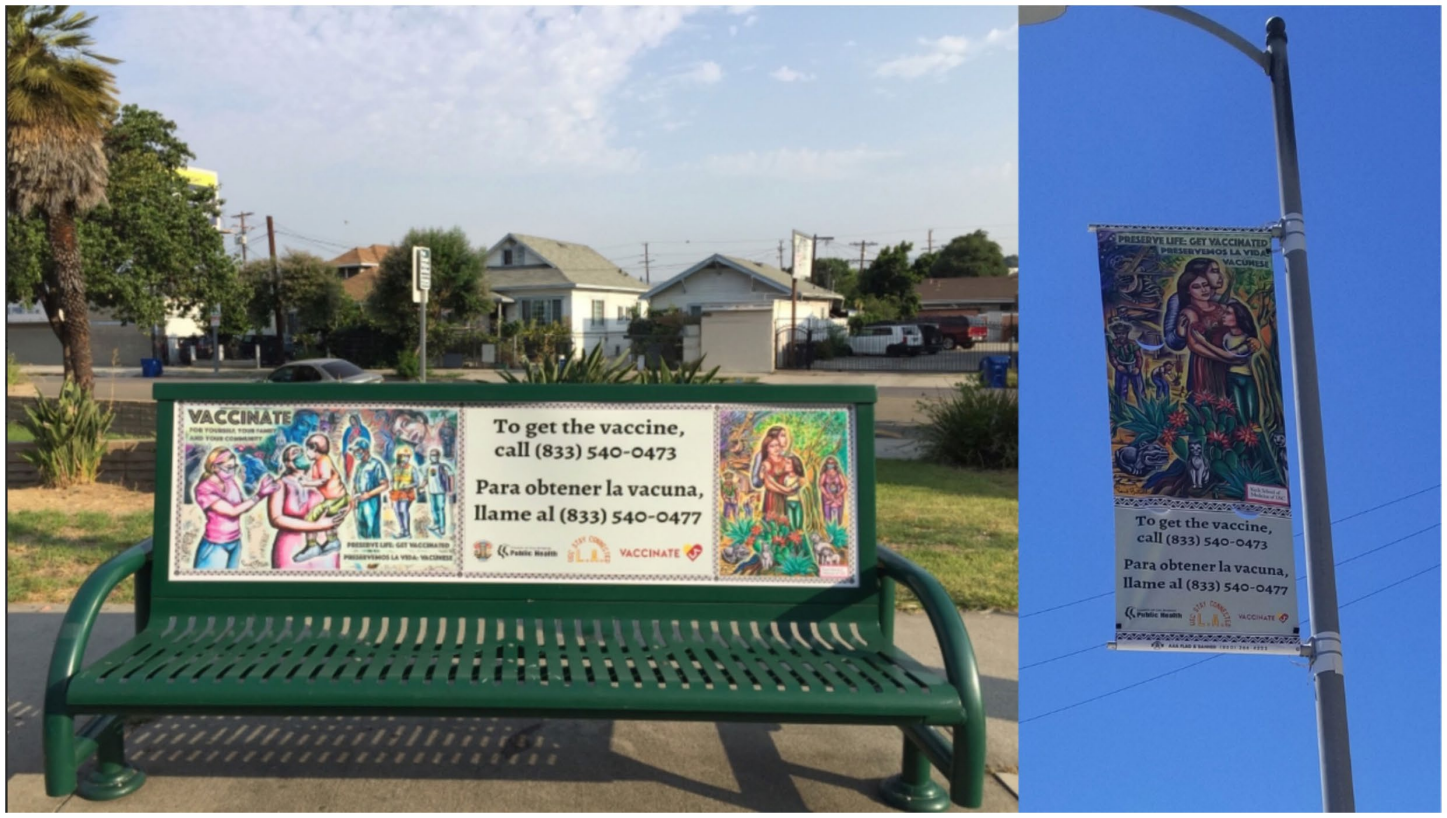
Stay connected Los Angeles outdoor campaign.

**Figure 2:**
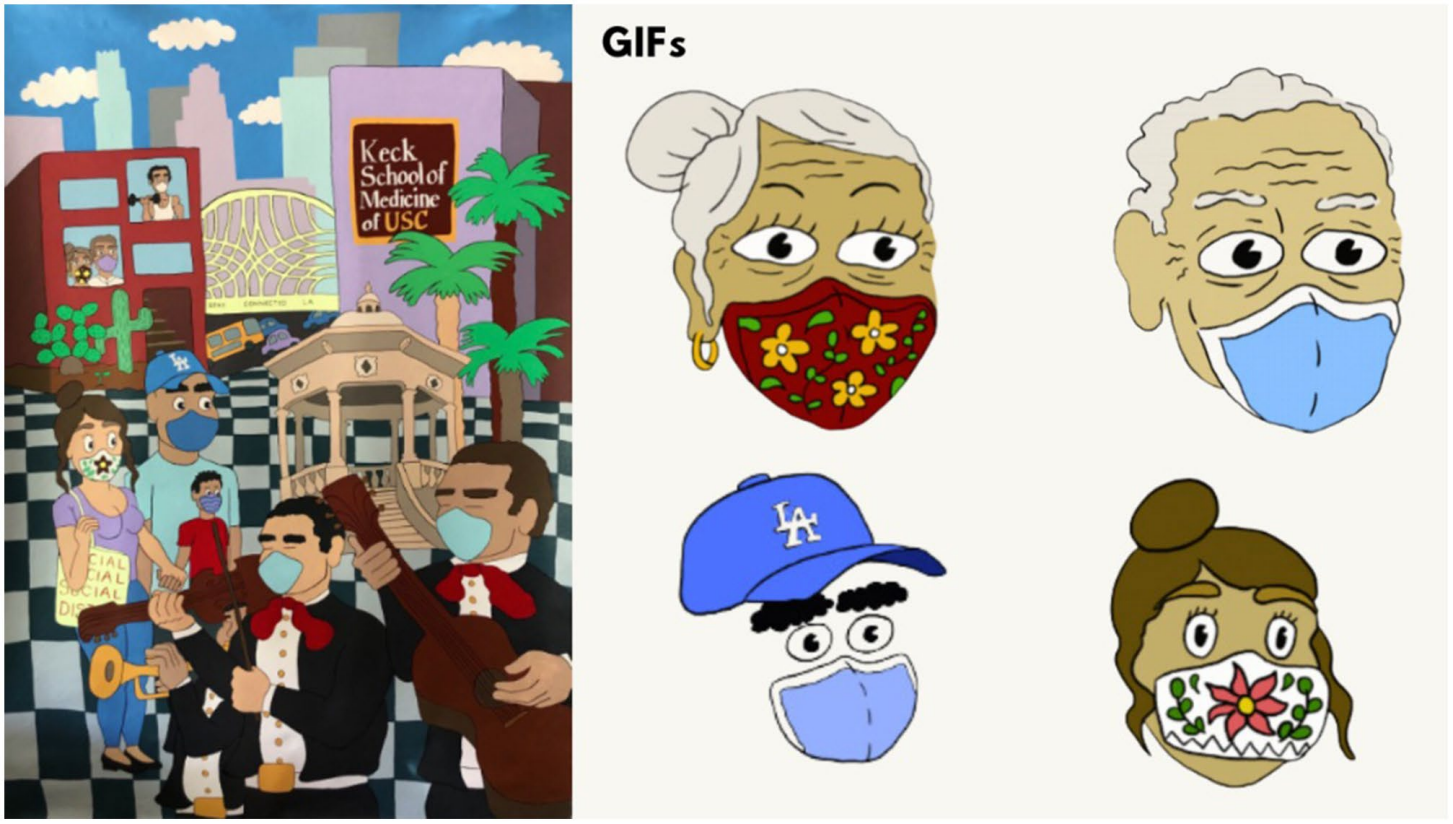
Social media GIFs and artwork created by Aaron Gonzalez.

**Figure 3:**
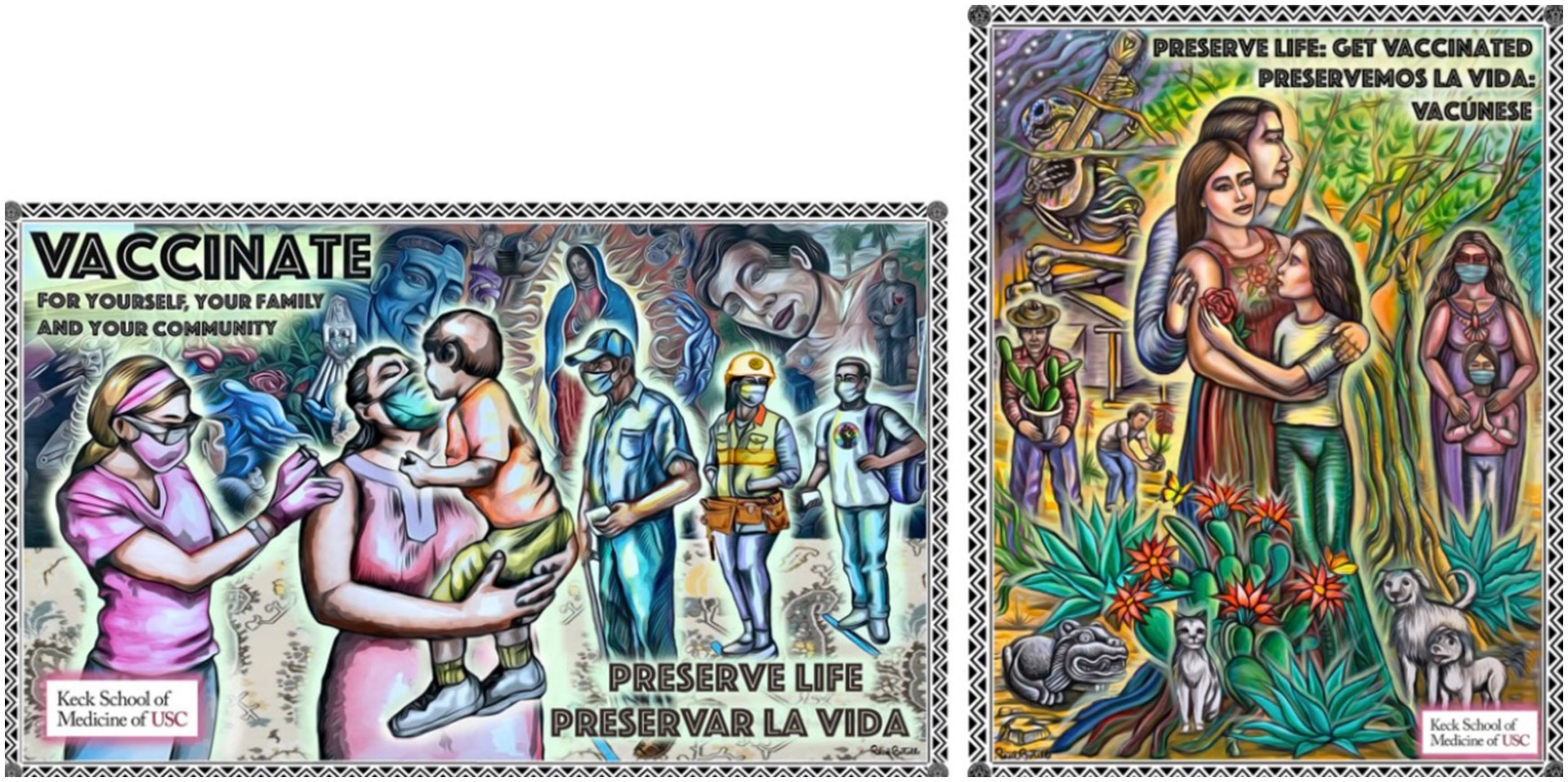
Preserve life: campaign murals created by Paul Botello.

**Figure 4:**
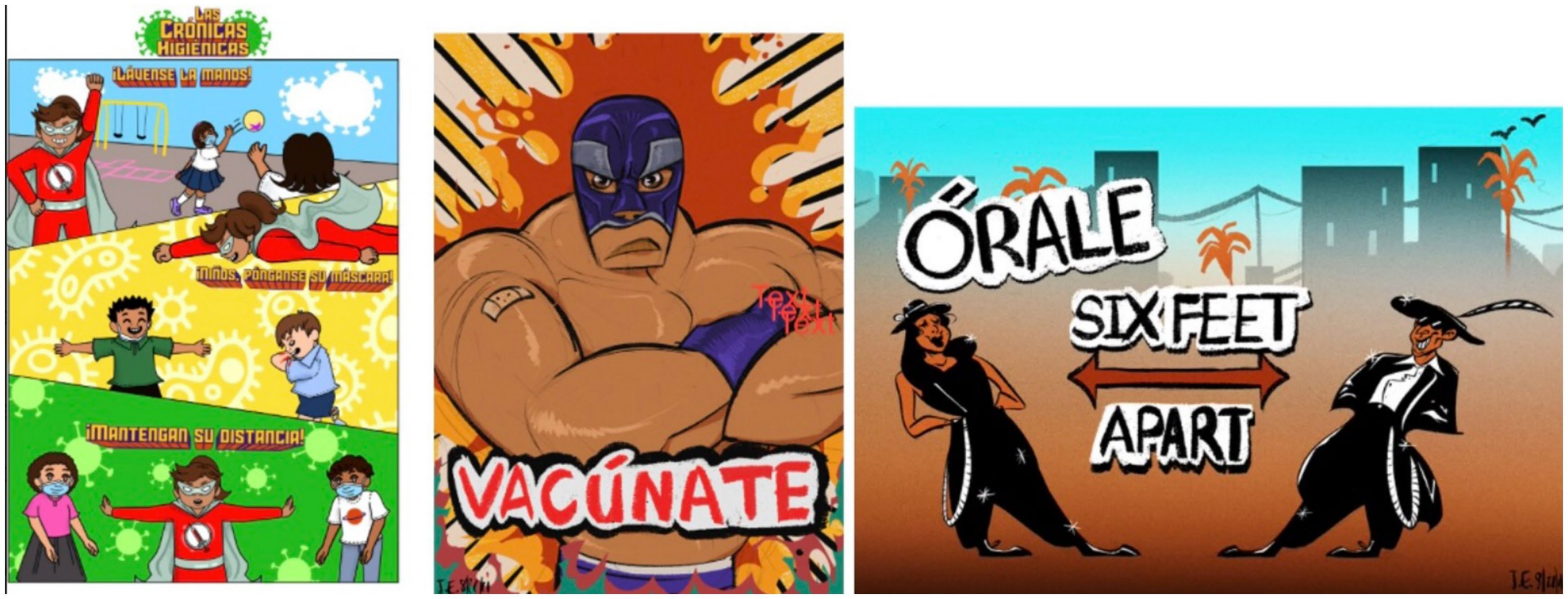
Spanglish/Bilingual cartoon created by J.E.

**Figure 5:**
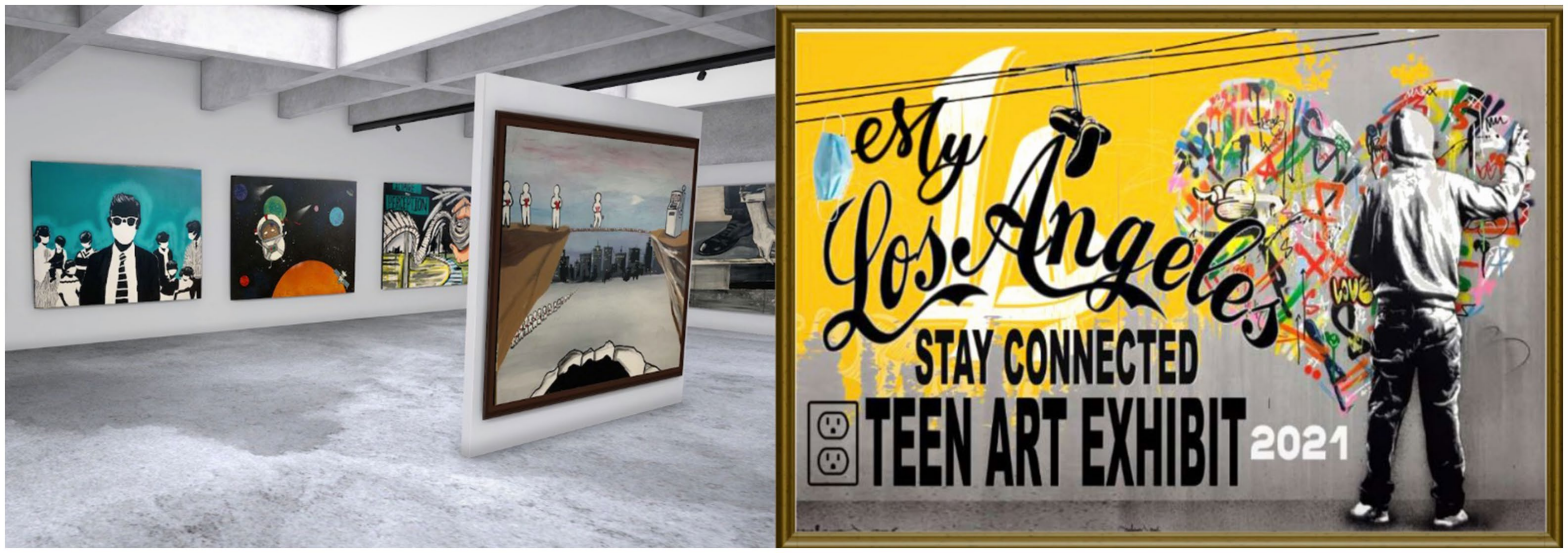
My Los Angeles Teen Art Online created by Ramona Gardens and Estrada Courts boys and girls club students.

In the accompanying (Figures 1–5), we provide images of the artworks included in the SCLA campaign. Monthly meetings with artists and public health specialists, alongside community input, provided ample opportunities for feedback in refining the campaign images and messaging. Participants voted on a main campaign image to be selected for display. Figure 1 depicts the main campaign images displayed on bus benches, billboards, and lamp posts in the Eastern part of Los Angeles.

### 3.2 Campaign elements: social media and GIFs

Discussion on the use of GIF animations for public health messages on social media revealed unanimous support from participants, with thematic analysis shown in Table 1. Participants highlighted the widespread use of GIFs, particularly among a younger demographic, emphasizing their effectiveness. Others echoed this sentiment, emphasizing that GIFs provide a quick and snappy way to convey health messages, making them accessible for younger individuals and those who might not be tech-savvy. The conversation then shifted to the representation of frontline workers, with participants expressing diverse opinions. Social Media elements and GIFs used in the SCLA campaign appear in Figure 2, and may be accessed online to view the animated GIFs.^2^ Participants emphasized that not all frontline workers are healthcare professionals and questioned the symbolism of the heart with a cross (see Figure 1), which may predominantly represent medical personnel. Others appreciated the inclusion of LA landmarks, suggesting that COVID affects people in various areas of the city. However, Participants stressed the importance of acknowledging essential workers beyond healthcare professionals. Participants proposed modifying the heart image to include a phrase about different frontline workers, and several participants highlighted the need for more representation of community assets.

**Table 1:** Participants’ response to social media GIFs.

Recurrent themes	Number of participants supporting (percentage endorsed each theme)
Representation of essential workers	6 (40%)
Lack of representation of community areas	4 (27%)
Focus on popular landmarks	5 (33%)
Message not serious enough	3 (20%)
More emphasis on family representation	4 (27%)
Clearer message for billboard	2 (13%)

Concerns were raised about the GIF artwork’s suitability for the Hispanic community, with participants expressing that it might cater more to tourists than local residents. Participants advocated for improvements, urging the artwork to be more representative of their communities, essential places like grocery stores, and a more serious message about the importance of staying at home and wearing masks. The consensus was that the current focus on iconic LA landmarks may convey the wrong message.

Despite these critiques, participants agreed overall that the artwork encourages people to wear masks and practice social distancing, which were mitigating behaviors selected to promote. Participants recommended the use of platforms like Instagram, X, TikTok, and Facebook for disseminating the artwork, citing its potential appeal to younger audiences. Other participants suggested specific improvements, emphasizing the need for greater community representation and a more serious tone to convey the gravity of the COVID-19 situation at the time, as the campaign was displayed prior to the widespread use of vaccines.

### 3.3 Postpone life to preserve life: campaign mural

The “Postpone Life to Preserve Life” mural proposal ignited rich discussions among participants (Figure 3), offering diverse perspectives on its thematic elements. Breakdown of thematic analysis for this piece is provided in Table 2. Crafted by a local artist, the mural depicted two scenes capturing the essence of pandemic life and the imperative of maintaining connections while prioritizing safety. The participants expressed admiration for the mural’s representation and beauty. Participants also commended the depiction of a healthcare worker and a single parent, emphasizing its resonance with the diverse demographics of the Eastern part of Los Angeles. Participants echoed a sentiment of relatability, noting how the images resonated with the Mexican and Latino identity, particularly within low-income communities.

**Table 2:** Participants’ response to postpone life to preserve life mural.

Recurrent themes	Number of participants supporting (percentage endorsed each theme)
Positive response to mural	9 (60%)
Adding older adult and masks to murals	8 (53%)
Community engagement and reflection	7 (47%)
Billboard, mural, lamppost preferences	7 (47%)
Colorful and attention grabbing murals	6 (40%)
Family, multigenerational component	6 (40%)
Reflection of COVID impact on communities	6 (40%)
Message clarity and self-explanatory	6 (40%)
Representation of vulnerable population	5 (33%)
Support for vaccination	4 (27%)

“These pictures, like all of us relate to them, as Mexican, as being Latinos, as being you know, wherever we are in our low income communities. It really hits home to most of us.”

“I feel like that represents a lot of moms in our communities, like they're doing a lot of multitasking, caring for their children in you know, on their arms, while also trying to comply for their own well-being and the well-being of their children.”

Another salient theme emerged in the discussions—the importance of multigenerational representation. Participants emphasized the significance of incorporating older adults into the mural. They highlighted the reality of multigenerational households and the need to address the vulnerabilities of the older adult during the pandemic. Concerns about educational content and realism were raised by some participants, noting the potential ambiguity of conveying preventive messages. The participants also engaged in a dialogue about the accessibility and community engagement potential of the mural, expressing preferences for placements such as bus stops and MTA spaces.

Overall, the participants’ opinions unveiled a shared appreciation for the aesthetic appeal and cultural resonance of the “Postpone Life to Preserve Life” mural. Their considerations underscored the importance of thoughtful representation, educational clarity, and strategic placement, revealing the mural’s potential as a potent instrument for public health messaging. The conversation highlighted the participants’ commitment to fostering unity and encouraging responsible behavior within the community as they navigate the ongoing challenges posed by the pandemic.

### 3.4 Spanglish project/bilingual cartoon

Visual messaging campaigns, instrumental in disseminating crucial information amid the COVID-19 pandemic, were subject to critical evaluation. One predominant theme surfaced as participants expressed uncertainty regarding Spanglish/Bilingual cartoon’s (Figure 4) relevance to COVID-19 (thematic analysis in Table 3). One participant explicitly questioned the connection, stating, “Does this have to do anything with COVID?” This sentiment resonated, with a subsequent remark firmly asserting, “No, it does not. It does nothing.” A participant emerged as the primary advocate for this theme, raising valid concerns about the project’s alignment with the overarching goal of a COVID-19 messaging campaign.

**Table 3:** Participants response to Spanglish COVID Cartoon.

Recurrent themes	Number of participants supporting (percentage endorsed each theme)
Relevance of proposed artwork to COVID	2 (13%)
Concerns about adding comedy to COVID theme	2 (13%)
Target audience and effectiveness of cartoons	3 (20%)
Nostalgic feeling and classroom setting	2 (13%)

A second theme centered around the potential pitfalls of infusing humor into COVID-19 messaging. Participants expressed apprehensions about the possible dilution of the seriousness of the pandemic message through comedic elements. One participant noted, “if you add comedy to COVID, it could be misleading and kind of not be taken as serious as it should be.” This theme unveils a nuanced discussion around the balance between engaging visual styles and the gravity of the subject matter.

We also explored the effectiveness of the cartoon visual style in targeting younger audiences. A younger participant supported the use of cartoons, emphasizing the need to convey the message effectively. She suggested that visual style is secondary, stating, “As long as they get the actual message across, it could be a superhero wearing a mask; it could be anything saying, ‘Oh, it’s important to wear a mask.’ I think that’s what really matters.” The difference in reception among younger and older participants in these focus groups reflects the importance of adapting visual campaigns to cater to the unique preferences and receptiveness of diverse age groups.

In the final theme, the discussion shifts toward audience segmentation and the potential advantages of targeting specific demographics. One participant highlighted the proposal’s potential resonance in a classroom teacher setting, capturing a nostalgic feeling. This theme underscores the multifaceted nature of crafting visual campaigns that not only educate but also evoke emotions and memories within specific communities.

In conclusion, Spanglish Project’s evaluation reveals nuanced perspectives on clarity, humor, audience targeting, and nostalgic appeal. These themes contribute to a deeper understanding of the challenges and considerations inherent in developing visual messaging campaigns that effectively resonate with diverse audiences amid the complexities of the ongoing COVID-19 pandemic.

### 3.5 My Los Angeles Teen Art Online Exhibit

The proposed theme of this exhibit, centering on youth engagement in creating artwork, featured several art pieces created by students of the Boys and Girls Club in Ramona Gardens and Estrada Courts (Figure 5; thematic analysis in Table 4). This garnered support from participants, emphasizing the importance of allowing young individuals to creatively interpret and express their feelings about COVID-19, envisioning it as a platform for them to communicate their experiences and coping mechanisms. The direct quote from one participant highlights the potential of this approach: “If they are thinking, like, I want to design something, because, at the same time, I want to keep myself safe and get my message, my creativity across to the public, I think it’s a good way.”

**Table 4:** Participants response to My Los Angeles Teen Art Exhibit.

Recurrent themes	Number of participants supporting (percentage endorsed each theme)
Importance of vaccination	3 (20%)
Consideration of community representation in art	3 (20%)
Clarity and Straightforwardness in art messages	2 (13%)
Cultural sensitivity in art	1 (6%)
Importance of clear education in art messages	1 (6%)

Another theme that emerged was the consideration of the target audience and representativity. Participants proposed that the exhibit might be more resonant with junior high or high school-aged individuals, suggesting that the messages might lack a family-oriented focus. One participant expressed reservations about the general effectiveness of the art pieces in the exhibit conveying a unique culturally tailored Hispanic specific message. This theme is captured by the direct quotes from a participant: “I think this is more for junior high or high school age; it does not really have a whole family-oriented message” and “The youth are the ones that know what messages work for them, so by having them do that, it’ll be able to represent what the world looks like for them.”

The final theme of the reviews of this exhibit centered on the artistic impact and educational content of the proposed artworks. Participants had varying views, with some recognizing the potential attention-grabbing aspect of some of the pieces, while others expressed a lack of personal connection and understanding of the gallery’s meaning. One participant emphasized the need for educational content and prevention messages, pointing out potential gaps in conveying crucial information during the COVID-19 pandemic. These perspectives highlight the multifaceted considerations in evaluating the proposed gallery’s effectiveness. The nuanced insights from the interview contribute to a preliminary understanding of the My Los Angeles Teen Art Online Exhibit, setting the stage for a more in-depth analysis in the ensuing research paper.

## 4 Discussion

### 4.1 Key themes

**Figure 6:**
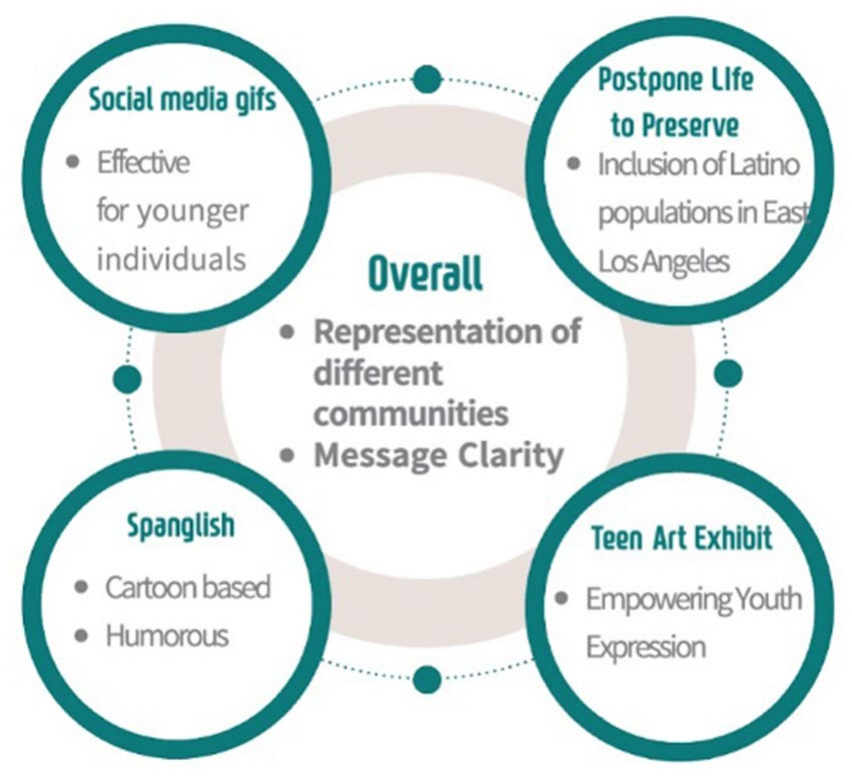
Stay connected Los Angeles major themes diagram.

In examining the four distinct COVID-19 visual campaign elements, several recurrent themes emerged from our analysis, illustrated in Figure 6. One recurring theme that resonated across projects was the promotion of youth engagement and creative expression. This theme underscores the vital role of allowing young individuals to interpret and express their feelings creatively, offering a unique lens through which to address the challenges posed by the pandemic or a public health emergency. The explicit emphasis on youth involvement in the My Los Angeles Teen Art Exhibit campaign element encapsulates the dedication to fostering a space where creative expression becomes a powerful conduit for conveying messages related to COVID-19 or other diseases or public health emergencies.

Another critical theme that became apparent is the importance of the target audience. Community participants contribute to a dialogue emphasizing the importance of tailoring visual messaging campaigns to specific age groups. The shared sentiment is that certain projects may resonate more strongly with junior high or high school-aged individuals. This theme underlines the significance of understanding and catering to the diverse age demographics within the community, recognizing that effective communication requires a nuanced approach tailored to the receptiveness of different age groups.

The essential role of educational content and clarity in conveying health education messages through art also emerged as a recurrent theme. Participants engaged in the evaluation of the “Spanglish/Bilingual Cartoon” and “Postpone Life to Preserve Life Mural” expressing concerns about the relevance and potential ambiguity in the proposed artworks. This theme accentuates the importance of clear artistic expressions, specific health messaging aligned with overarching health education goals, especially in projects aimed at disseminating crucial information during a public health crisis.

These themes collectively underscore the intricate layers of perspective present in the evaluation of visual messaging campaigns related to COVID-19 with wide implications for art meets health future public health community education. While the encouragement of youth engagement and creative expression stands out prominently, considerations about the target audience, cultural expressions, age related art preferences, educational content, and community representation illuminate the nuanced nature of crafting effective visual campaigns that incorporate both art and health messaging. This thematic analysis provides insights into how ongoing health education campaigns can be improved and offers valuable considerations for the development of future visual messaging strategies that mitigate the impact of the COVID-19 pandemic, future emergent diseases, and public health emergencies on Hispanic populations and other vulnerable communities.

### 4.2 Lessons learned and conclusion

When developing future public health campaigns, especially those that bring arts and health together, it is crucial to emphasize communal connection and the clarity of health messages and visuals. These elements serve as vital tools for educating the community and reinforcing behaviors that enhance public health. Our findings indicate a particularly positive response to mural art, suggesting that incorporating mural-based artwork focused on health promotion and disease prevention could significantly benefit future campaigns. Art and creative mediums can serve as useful tools against social isolation while also facilitating emotional expression. During the COVID-19 pandemic, engagement with art and creative activities was linked to improved mental well-being and a stronger sense of social connection. This underscores the value of integrating artistic elements into public health strategies (16).

For the dissemination of health messaging through art, strategic placement in high-traffic areas is essential. Locations such as tourist spots, healthcare facilities, gas stations, grocery stores, and areas near local street vendors are ideal for maximizing visibility and impact. Additionally, community billboards, bus benches, and lamp posts in frequented locations can significantly enhance the campaign’s reach within the community.

Leveraging social media platforms such as X, Instagram, TikTok, and other applications has significant potential to extend the reach of public health campaigns. Content designed in Spanish or bilingual formats are likely to be particularly effective in engaging diverse audiences.

The credibility of a public health message greatly depends on its spokesperson. Local community members and medical professionals, who are well-known and trusted, were found in this study to be the most effective in conveying health education messages that merge arts and health. In an era where scientific information often faces skepticism and misinformation is rampant, these trusted voices play a crucial role in maintaining message credibility and encouraging healthy behaviors. Our research highlights the significant role of community health workers and promotors de salud as trusted sources of information in the Hispanic community. Aligning with existing literature, leveraging local Hispanic community leaders as spokespersons for health promotion and disease prevention is highly effective in Hispanic communities (7).

Identification of and partnership with community leaders can be a challenge for health organizations. This arts-meets-health campaign benefitted from longstanding partnerships that have been established between our institution and our neighboring communities throughout Eastern Los Angeles. To maximize community engagement, trust, and retention, we recommend establishing long-term partnerships with community organizations to address a network of issues that are of interest to both health institutions and the community. Examples of community organizations that have successfully partnered with health institutions on preventive health campaigns are diverse and include churches, barbershops, nail salons, and recreation centers (17, 18). Such partnerships require concerted leadership from both the community and health institution to maintain communication, for example through the monthly community advisory board meetings that underpinned this campaign. Further, community members must be adequately compensated for their time and expertise in these meetings, necessitating appropriate funding allocation. For long-term participation as a community advisory board member, this compensation may amount to $1,000/year depending on responsibilities, whereas participants in individual surveys or focus groups may be remunerated a modest gift card amount for their time. In the context of public health campaigns, these investments are comparatively modest, while yielding benefits in improved reception and behavior change.

Such investment in community input benefitted this campaign through the revision and selection of our COVID-19 messaging, yielding positive participant feedback. The crafting of clear, culturally sensitive messages provided in language that considers the diversity and heterogeneity of the Hispanic population is essential. Tailoring messages to accommodate age differences further ensures the relevance and resonance of health campaigns within diverse communities.

The insights from participants underscore the value of integrating impactful health education messaging with art to effectively reach and engage the heterogeneous Hispanic communities. This approach not only provides a platform for local artists to contribute to community health and safety creatively but also exemplifies the benefits of community-based participatory research. Looking ahead, employing similar artistic and community-focused strategies can effectively target historically marginalized or disproportionately affected groups in public health initiatives.

### 4.3 Limitations

The inherent subjectivity of the participants’ responses must be considered. Participants were encouraged to share personal ideas, thoughts, and feelings. Additionally, external factors relating to the context of the focus group discussion, such as time of day, life events, fatigue, or mood could have further affected participant responses.

The subjectivity of the coders’ analyses must also be considered. The interpretation of written language and speech perceptions can significantly vary among individuals, influenced by their unique life experiences. This aspect may have contributed to systematic differences between the coders’ interpretation of qualitative data. We aimed to ensure quality coding by having both coders complete their analyses independently. Nevertheless, the coordination of the two coders provided important insights into what was similar and what differed in our findings.

Moreover, the qualitative nature of our data, collected in the Eastern part of Los Angeles with community participants, limits the generalizability of our findings to other Hispanic populations. Future studies should account for age differences, language differences, and differences by acculturation.

We recommend that our arts-meets-health and community-based approach could be used to assist other traditionally overlooked communities, such as the unhoused and people who use intravenous drugs or other substances. A previous study (19) highlighted the utility of introducing an arts-meets-health campaign in unhoused communities. Participants were able to create art pieces that were both personally and collectively meaningful, and develop positive social connections and support systems to mitigate the effects of social isolation. The use of art as a creative outlet also enabled many participants to have a motivation to lessen or halt problematic drug use, further illustrating the utility of this campaign in unhoused communities and with people who use intravenous drugs or other substances. Another intervention (20) also highlighted the utility of an arts-based public health campaign in communities that are affected by houselessness and intravenous drug use. This study determined that artistic-based health messaging and campaigning was one of the most impactful interventions for increasing feelings of social connectedness and knowledge regarding drug use safety awareness. From the two studies, it is clear that unhoused communities and people who use intravenous drugs and other substances can clearly benefit from an arts-based health intervention to improve feelings of social connectivity and promote health safety behaviors.

## Data availability statement

The original contributions presented in the study are included in the article/supplementary material, further inquiries can be directed to the corresponding author.

## Ethics statement

The studies involving humans were approved by Institutional Review Board (IRB) - University of Southern California. The studies were conducted in accordance with the local legislation and institutional requirements. The participants provided their written informed consent to participate in this study. Written informed consent was obtained from the individual(s) for the publication of any potentially identifiable images or data included in this article.

## Author contributions

TU: Conceptualization, Formal analysis, Writing – original draft, Writing – review & editing. WL: Formal analysis, Writing – original draft, Writing – review & editing. JP: Project administration, Supervision, Writing – original draft, Writing – review & editing. AB: Writing – original draft, Writing – review & editing. RB: Data curation, Writing – review & editing, Project administration. LB-G: Conceptualization, Funding acquisition, Investigation, Project administration, Resources, Supervision, Writing – original draft, Writing – review & editing.

## Footnotes

^1^ https://stayconnectedla.com

^2^ https://www.stayconnectedla.com/artistprojects/aarongonzalez
